# A new species of *Acorhinotermes* Emerson, 1949 (Blattodea, Isoptera, Rhinotermitidae) from Colombia, with a key to Neotropical Rhinotermitinae species based on minor soldiers

**DOI:** 10.3897/zookeys.891.37523

**Published:** 2019-11-21

**Authors:** Daniel Castro, Rudolf H. Scheffrahn

**Affiliations:** 1 Instituto Amazónico de Investigaciones Científicas SINCHI, Avenida Vásquez Cobo Calles 15 y 16, Leticia, Amazonas, Colombia Instituto Amazónico de Investigaciones Científicas SINCHI Leticia Colombia; 2 Fort Lauderdale Research and Education Center, Institute for Food and Agricultural Sciences, University of Florida, 3205 College Avenue, Davie, Florida 33314, USA University of Florida Davie United States of America

**Keywords:** Colombian Amazon, *
Dolichorhinotermes
*, *
Rhinotermes
*, taxonomy, termites

## Abstract

*Acorhinotermes* Emerson, 1949 is the only Neotropical Rhinotermitine genus with no major soldier. Herein *Acorhinotermes claritae* Castro & Scheffrahn, **sp. nov.** is described based on minor soldiers and an alate nymph collected in a secondary rain forest in the Colombian Amazon. The minor soldier of *A. claritae* Castro & Scheffrahn, **sp. nov.** has longer mandibular points and it is comparatively smaller than *A. subfusciceps*. An illustrated key to the minor soldiers of the Neotropical species of Rhinotermitinae is presented.

## Introduction

The subfamily Rhinotermitinae Froggatt, 1897 comprises worldwide the genera *Parrhinotermes* Holmgren, 1910, *Macrorhinotermes* Holmgren, 1913, *Schedorhinotermes* Silvestri, 1909, *Rhinotermes* Hagen, 1858, *Dolichorhinotermes* Snyder & Emerson, 1949, and *Acorhinotermes* Emerson, 1949. The last three genera are recorded from the Neotropical region (Maiti 2011; Krishna et al. 2013).

*Acorhinotermes subfusciceps* was originally described within the genus *Rhinotermes* (Emerson 1925; Snyder 1949). Emerson in Snyder (1949) transferred *Rhinotermes subfusciceps* to a new genus, *Acorhinotermes*, straightening the absence of the major soldier as a diagnostic characteristic of the genus.

Colombia has very few records of species of the subfamily Rhinotermitinae, only *Rhinotermes hispidus* Emerson, 1925 and *Rhinotermes marginalis* (Linnaeus, 1758) are reported (Pinzón et al. 2017; Constantino 2019). *Acorhinotermes* has been reported for Brazil, Guyana, French Guiana, Venezuela and Peru (Snyder 1949; Davies et al. 2003; Salick et al. 2013; Scheffrahn unpubl. data; Silva et al. 2019). Currently, only *Dolichorhinotermes japuraensis* Constantino, 1990 is endemic to the Amazon Basin (Constantino 1991). Additionally, all Rhinotermitinae species except *Dolichorhinotermes longidens* (Snyder, 1924) are found in the Amazon Region (Castro unpubl. data, Constantino 1992, Constantino and Cancello 1992, Krishna et al. 2013).

In this paper, we describe a new species *Acorhinotermes claritae* sp. nov. based on characters from the minor soldier and alate nymph. We provide as well as an illustrated key for the Neotropical Rhinotermitinae based on the minor soldier caste, which would be very helpful when major soldiers or imagoes are not represented in the collected samples.

## Materials and methods

Specimens of *Acorhinotermes claritae* sp. nov. were collected in trucks of dead trees with aspirators, at weet season (July 12–19, 2018), in the southern state of Amazonas, Colombia, and preserved in 95% ethanol. The holotype and paratypes are deposited in the “Colección de Artrópodos Terrestres de la Amazonía Colombiana”, SINCHI Amazon Institute of Scientific Research, Leticia, Amazonas, Colombia (**CATAC**). Paratypes are also deposited in the Termite Collection, Fort Lauderdale Research and Education Center, University of Florida, Davie, Florida, United States of America (**UF**).

Additional material examined for the Rhinotermitine species key is deposited in the UF and the CATAC, as follows: *Acorhinotermes subfusciceps*, PERU, (-9.05222, -75.57818), 30/05/2014, R. Scheffrahn col., 376 m (PN.799.0); *Dolichorhinotermes lanciarius* Engel & Krishna, 2007, PERU, (-11.06414, -74.71955), 25/05/2014, R. Scheffrahn col., 602 m (PN.104.0); *Dolichorhinotermes longidens*, PANAMA, (9.34349, -79.77382), 4/06/2005, R. Scheffrahn col., 216 m (PN.684.0); *Dolichorhinotermes longilabius* (Emerson, 1924), FRENCH GUYANA, (5.03784, -52.95580), 7/02/2008, J. Krêcêk col., 87 m (FG.181.0); *Rhinotermes hispidus*, BOLIVIA (-16.99937, -65.62736), 26/05/2013, R. Scheffrahn col., 491 m (BO. 163.0); *Rhinotermes marginalis*, BOLIVIA, (-16.97043, -65.21001), 26/05/2013, col. R. Scheffrahn, 231 m (BO. 76.0); *Dolichorhinotermes longilabius*, COLOMBIA, (4.343416, -69.98627), col. L. Pinedo, 101 m (CATAC-03314); *Rhinotermes hispidus*, COLOMBIA (3.8210, -67.81041), 16/03/2019, col. J. Chase, 98 m (CATAC-03687); *Rhinotermes marginalis*, COLOMBIA, (-3.80044, -70.31533), col. J. Chase, 76 m (CATAC-03558). In the other hand, Emerson (1925), Constantino (1990), Snyder (1924), Snyder (1926) and Desneux (1904) were consulted for those species not represented in the UF or CATAC collections.

Morphological characters used for the alate nymph and minor soldier follows Roonwal (1970). Microphotographs were taken as multi-layer montages using a Leica M205C stereomicroscope controlled by Leica Application Suite version 3 software. Preserved specimens were suspended in a pool of Purell Hand Sanitizer to position the specimens on a transparent Petri dish background.

## Taxonomy

### Acorhinotermes claritae

Taxon classificationAnimaliaBlattodeaRhinotermitidae

Castro & Scheffrahn sp. nov.

54D29676-EE5C-5DC5-815A-C7687FE70D22

http://zoobank.org/92519036-1333-4086-9485-BBF1D8A33B3E

#### Type material.

***Holotype*.** Minor soldier from colony CATAC 2722.

#### Type-locality.

COLOMBIA: Amazonas, Leticia (-4.08975, -69.92705).

***Paratypes*.** COLOMBIA, Amazonas, Leticia, (-4.08975, -69.92705): 12.VII.2018, James Chase col., 87 m, 1 alate nymph, 45 minor soldiers, 156 workers (CATAC 2722); 12.VII.2018, Daniela Manso col., 87 m, 11 minor soldiers, 56 workers (CATAC 2723); (-4.08900, -69.92497): 12.VII.2018, James Chase col., 91 m, 5 minor soldiers, 2 workers (CATAC 2724); (-4.04875, -70.00527): 13.VII.2018, Daniela Manso col., 106 m, 33 minor soldiers, 41 workers (CATAC 2750); (-4.04972, -69.92704): Daniel Castro col, 97 m, 5 minor soldiers, 4 workers (UF no. CO 918).

#### Diagnosis.

Minor soldier head with concave lateral margins forming a posterior constriction, with prominent mandibular points extend beyond the fontanelle.

#### Description.

**Figure 1.**
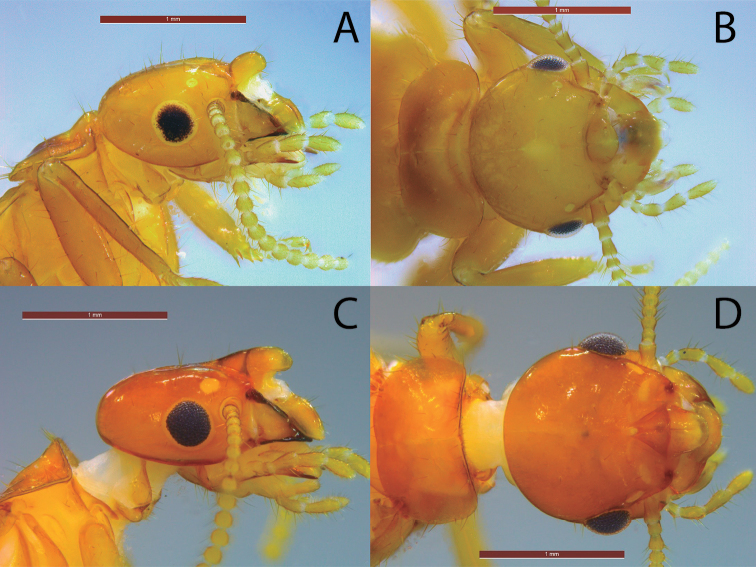
*Acorhinotermes* spp. **A, B** alate nymph of *Acorhinotermes claritae* sp. nov., lateral and dorsal view **C, D** imago of *Acorhinotermes subfusciceps*, lateral and dorsal view. Scale bar: 1 mm.

***Alate nymph*.** (Fig. 1A, B) Head capsule yellowish-brown, widely oval with numerous long bristles. Antennae with 20 articles, 2<3=4. Dorsum of body concolorous with head capsule. Compound eyes subcircular, eye margins wide and broadly separated from antennal sockets. Ocelli of small size, oval, well separated from eyes. Clypeus linguiform, not buttressed by frontal projection. Pronotum margin with numerous long bristles; rounded lateral margins. Mandibles with M1 more prominent than apical teeth. Right mandible with M1 more projected than left mandible. Left mandible with M2 projected to half the length of M1, M2 and M3 forms an obtuse angle, M3 and molar tooth projected at same distance.

Measurements (mm) for a single alate nymph: head length with labrum 1.27, head length to postclypeus 1.46, maximum width of the head with eyes 1.39, width of head without eyes 1.21, diameter of eye 0.25, ocellus diameter 0.08, length of pronotum 0.78, width of pronotum 1.36, total body-length without wings 6.81.

***Comparisons*.***Acorhinotermes claritae* sp. nov. has more abundant bristles in lateral view. The ocelli and eyes are smaller than the *A. subfusciceps* imago, and the clypeal projection projects more acutely in *A. claritae* sp. nov. and it is not buttressed by a frontal projection as in *A. subfusciceps* (Fig. 1C).

**Figure 2.**
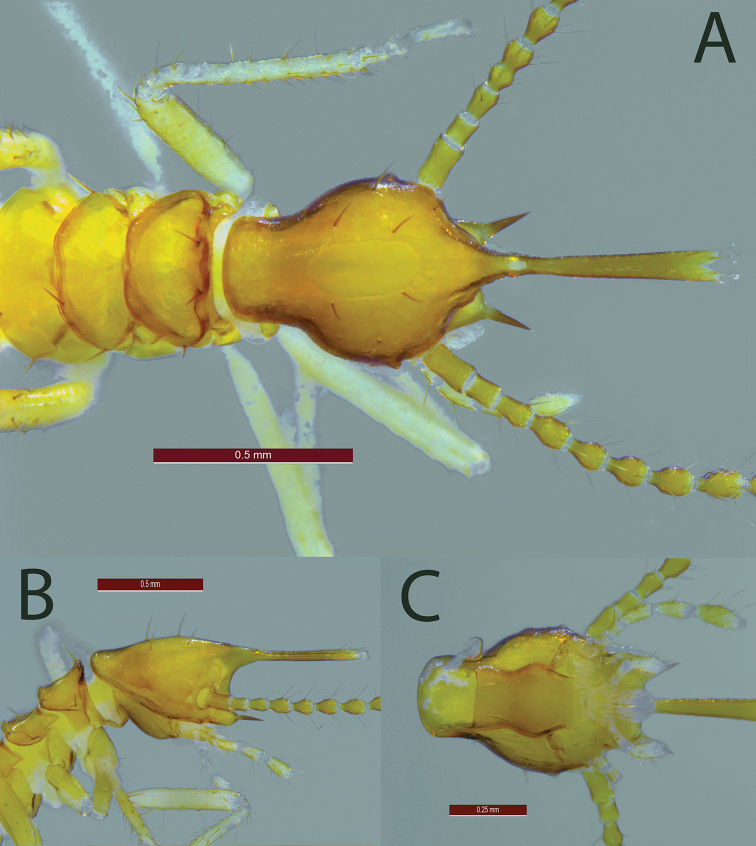
*Acorhinotermes claritae* sp. nov., minor soldier **A** head in dorsal view **B** head in lateral view **C** head in ventral view.

***Minor soldier*.** (Fig. 2; Table 1) Head capsule, in dorsal view, with concave lateral margins forming posterior constriction 10–12 long erect bristles, without microscopic hairs. Antennae with 15 or 16 articles, formula 2=3<4=5. Mandible vestigial, point long, straight and sharp. Labrum hyperelongate, broadening apically; tip bilobed; nearly in same plane as vertex in lateral view. Fontanelle at basal one-fifth of labrum. Pronotum concolorous with head, with 4–8 dispersed bristles, 2–4 in anterior margin and 2–4 in surface, pronotum without microscopic hairs. Tergites pale yellow, margins covered by dense layer of hairs. Legs with many long and short bristles; thick bristles on foretibia.

**Table 1.** Measurements (mm) of 10 minor soldiers from three colonies of *Acorhinotermes claritae* sp. nov.

	**Holotype**	**CATAC 2722**		**CATAC 2723**		**CATAC 2724**	
		**Range**	**Mean** ± **SD**	**Range**	**Mean** ± **SD**	**Range**	**Mean** ± **SD**
Max head width	0.53	0.45–0.58	0.53±0.05	0.53–0.63	0.58±0.03	0.55–0.66	0.59±0.03
Length head with labrum	1.30	1.18–1.36	1.31±0.06	1.23–1.38	1.32±0.05	1.23–1.40	1.28±0.05
Length of labrum	0.66	0.57–0.68	0.63±0.05	0.59–0.73	0.66±0.07	0.60–0.69	0.63±0.03
Pronotum width	0.42	0.39–0.46	0.43±0.03	0.43–0.49	0.47±0.02	0.44–0.56	0.48±0.04
Pronotum length	0.28	0.24–0.33	0.29±0.03	0.25–0.37	0.31±0.03	0.29–0.34	0.31±0.02
Length of hind tibia	0.84	0.81–0.89	0.85±0.03	0.77–0.88	0.82±0.03	0.80–0.93	0.84±0.04

**Figure 3.**
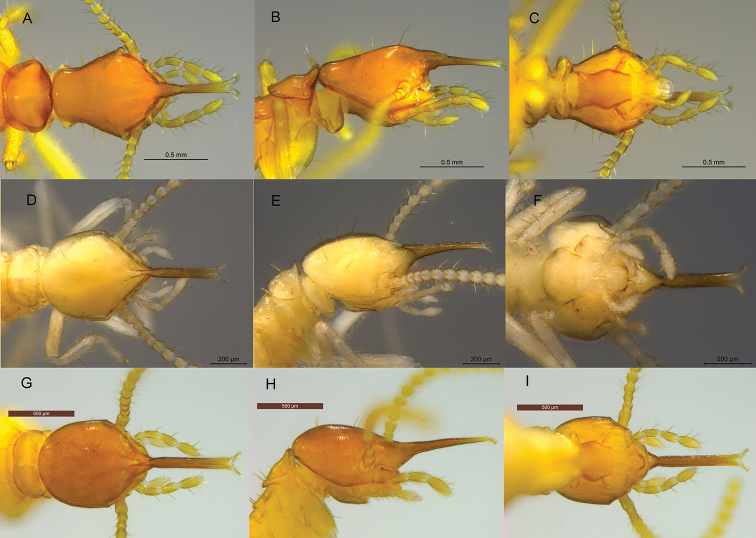
Minor soldiers of Neotropical Rhinotermitinae deposited in UF and CATAC termite collections **A–C***Acorhinotermes subfusciceps***D–F***Dolichorhinotermes longidens***G–I***Dolichorhinotermes lanciarius*.

***Comparisons*.***Acorhinotermes claritae* sp. nov. is smaller and has longer mandibular points than *A. subfusciceps*. In profile, the dorsa of the occiput, vertex, and labrum of *A. claritae* sp. nov. form a nearly straight line, while in *A. subfusciceps* this profile forms an obtuse angle (Fig. 3A, B). All minor soldiers of *Dolichorhinotermes* and *Rhinotermes* have the labrum tip bifurcated (forked or divided into two parts or branches), and it is much more bilobed in *A. subfusciceps* than in *A. claritae* sp. nov.

**Figure 4.**
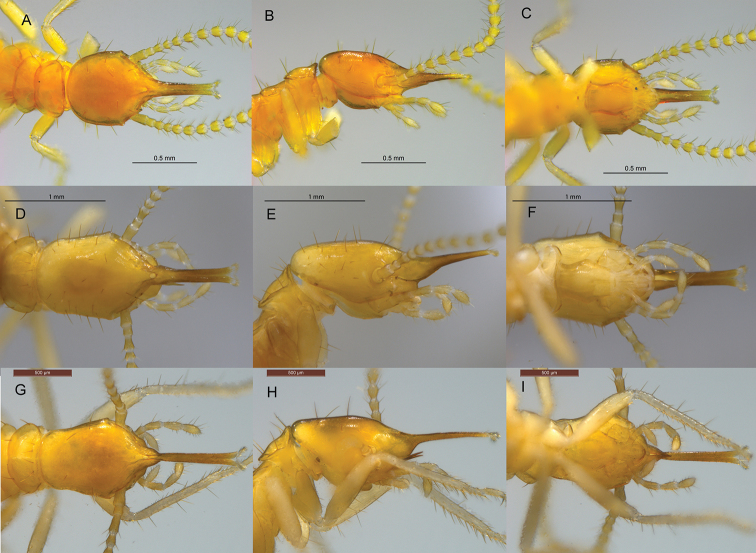
Minor soldiers of Neotropical Rhinotermitinae deposited in UF and CATAC termite collections **A–C***Dolichorhinotermes longilabius***D–F***Rhinotermes hispidus***G–I***Rhinotermes marginalis*.

#### Biological notes.

*Acorhinotermes claritae* sp. nov. was collected in a secondary rain forest near the Tacana river, close to a “chagra” (indigenous agricultural production system). During the wet season, these areas are in flood zones. The colonies were found in trunks of dead trees and in big dry branches on the ground. One particular colony of this species was found in a same dead branch together with *Heterotermes tenuis* (Hagen, 1858) and *Cylindrotermes parvignatus* Emerson, 1949, and another colony with *Silvestritermes gnomus* (Constantino, 1991). *Acorhinotermes claritae* sp. nov. was collected in a unique locality near the city of Leticia, although we did surveys in other two sites of a radius no greater than 15 km, it was not collected.

#### Distribution.

**Figure 5.**
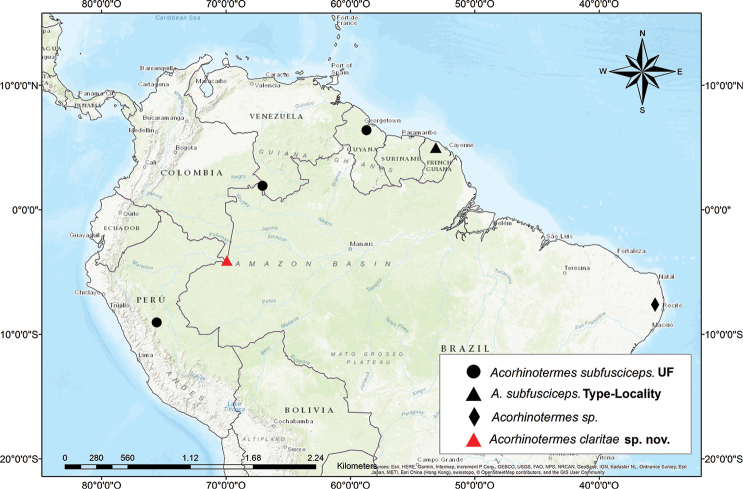
Distribution map of *Acorhinotermes* spp. Black circles are records from University of Florida termite collection and the black diamond is the record of Silva et al. (2019).

The genus *Acorhinotermes* is distributed in the Amazon basin, Guiana shield and Caatinga (Fig. 5). *A. claritae* sp. nov. is restricted to the Amazon basin.

#### Etymology.

The species is named in honor of Dr. Clara (Clarita) Peña-Venegas, who has supported and promoted the knowledge and inventories of termites and other terrestrial arthropods from the Colombian Amazon in the SINCHI Institute.

##### Key to the species of Neotropical Rhinotermitinae based on minor soldiers ^1^

**Table:** 

1	In dorsal view, fontanelle anterior to mandibular lobes (bases of mandibular points) (Figs 2B, 3A) (*Acorhinotermes*)	**2**
–	In dorsal view, fontanelle at or posterior to mandibular lobes (Figs 3D, 4A, D)	**3**
2	Mandibular points extend beyond the fontanelle (Fig. 2A)	***A. claritae* sp. nov.**
–	Mandibular points do not extend beyond the fontanelle (Fig. 3A–C)	***A. subfusciceps***
3	Mandibles points reduced to minute points on basal lobes (Fig. 3F, I)	**4**
–	Long mandible points prominent, directed upward and forward (Fig. 4B, D, H)	**5**
4	Smaller species: head length less than 1.10 mm. Panama (Fig. 3D–F)	***D. longidens***
–	Larger species: head length more than 1.47 mm. South America (see Engel and Krishna 2007: fig. 2)	***D. lanciarius***
5	Length of head to tip of labrum 1.35 mm or less	**6**
–	Length of head to tip of labrum 1.45 mm or more	**7**
6	Middle of anterior margin of pronotum with numerous short bristles, small mandibles do not exceed the base of the labrum, not visible from the dorsal view (see Constantino 1990: fig. 8)	***D. japuraensis***
–	Middle of anterior margin of pronotum smooth, without numerous short bristles, large mandibles reaching up to the middle of the labrum, visible from the dorsal view (Fig. 4A–C)	***D. longilabius*^2^**
7	In lateral view, about 4–10 setae visible on vertex, labrum very elongated and narrow with a slight depression at its base (Fig. 4G)	**8**
–	In lateral view, about 20–30 setae visible on vertex, labrum elongated and width without depression at its base (Fig. 4D)	**9**
8	Head in dorsal view with a defined constriction behind antennae (Fig. 4G)	***R. marginalis* , *R. nasutus*^3^**
–	Head in dorsal view without constriction behind antennae, posterior margin of the head rounded (see Emerson 1925: fig. 42C)	***D. tenebrosus***
9	Head length to labrum tip 1.70–1.93 mm (Fig. 4D–F)	***R. hispidus***
–	Head length to labrum tip 2.20–2.35 mm (See: Snyder (1926). Plate 1, fig. 2)	***R. manni***

## Discussion

In our Colombian survey, about 102 minor soldiers were collected without a single major soldier reinforces our belief that the latter caste is absent from *A. claritae* sp. nov. Among genera of the subfamily Rhinotermitinae, *Dolichorhinotermes* has been the most common in the Amazon region surveys, followed by *Rhinotermes* and then *Acorhinotermes* (Castro unpubl. data; Constantino 1991; de Souza and Brown 1994; Palin et al. 2011). However, *R. marginalis* is found in the West Indies while *D. longilabius* has not been reported from there, with the exception of the islands of Trinidad and Tobago where the latter species is common and the former has not been collected (Scheffrahn unpubl. data). *A. claritae* sp. nov. is the first record for the genus in Colombia.

In the key to genera of Neotropical termites, Constantino (2002) differentiated *Dolichorhinotermes* minor soldiers from *Rhinotermes* minor soldiers by the length of the head to the tip of the labrum, less than 1.2 mm, but *D. tenebrosus* and *D. lanciarius* measures greater than 1.2 mm. To differentiate these genera, the major soldier or imago caste is preferred. Major soldiers of *Dolichorhinotermes* have a narrow long labrum reaching near tips of mandibles while the major soldiers of *Rhinotermes* have a short wide labrum that extends no more than half the length of the mandibles when extended. Also, the known imagos of *Dolichorhinotermes* head width range is 1.18–1.29 mm while the *Rhinotermes* imago head width range is 1.90–2.18 mm.

## Supplementary Material

XML Treatment for Acorhinotermes claritae

## Footnotes

The key does not include the fossil species *Dolichorhinotermes apopnus* Engel & Krishna, 2007, *Dolichorhinotermes dominicanus* Schlemmermeyer & ancello, 2000, and *Rhinotermes miocenicus* Nel & Paicheler, 1993.

The species *Dolichorhinotermes latilabrum* (Snyder, 1926) and *Dolichorhinotermes neli* Ensaf & Betsch, 2002 were not included in this key because we suspect they are junior synonyms of *Dolichorhinotermes longilabius* (Emerson, 1924).

These species cannot be separated only with the minor soldier. It is necessary the imago or the major soldier caste.

## References

[B1] Constantino R (1990) Two new species of termites (Insecta, Isoptera) from western Brazilian Amazonia. Boletim do Museu Paraense Emílio Goeldi 6: 3–9.

[B2] Constantino R (1991) Termites (Isoptera) from the lower Japurá River, Amazonas State, Brazil. Boletim do Museu Paraense Emílio Goeldi 7: 189–224.

[B3] Constantino R (1992) Abundance and diversity of termites (Insecta: Isoptera) in Two Sites of Primary Rain Forest in Brazilian Amazonia. Biotropica 24: 420–430. 10.2307/2388613

[B4] Constantino R (2002) An illustrated key to Neotropical termite genera (Insecta: Isoptera) based primarily on soldiers. Zootaxa 67: 1–40. 10.11646/zootaxa.67.1.1

[B5] Constantino R (2019) Termite Database. University of Brasília. http://www.termitologia.net/termite-database [June 18, 2019]

[B6] Constantino R Cancello EM (1992) Cupins (Insecta, Isoptera) da Amazônia Brasileira: distribuição e esforço de coleta. Revista Brasileira de Biologia 52: 401–413.

[B7] Davies RG Hernández LM Eggleton P Didham RK Fagan LL Winchester NN (2003) Environmental and spatial influences upon species composition of a termite assemblage across Neotropical forest islands. Journal of Tropical Ecology 19: 509–524. 10.1017/S0266467403003560

[B8] Desneux J (1904) Notes termitologiques. Annales de la Société Entomologique de Belgique 48: 146–151.

[B9] Emerson AE (1925) The termites of Kartabo, Bartica District, British Guiana. Zoologica 6: 291–459.

[B10] Engel MS Krishna K (2007) New *Dolichorhinotermes* from Ecuador and in Mexican Amber (Isoptera: Rhinotermitidae). American Museum Novitates 3592: 1–8. 10.1206/0003-0082(2007)3592[1:NDFEAI]2.0.CO;2

[B11] Krishna K Grimaldi DA Krishna V Engel M (2013) Treatise on the Isoptera of the world: 2. Basal Families. Bulletin of the American Museum of Natural History 377: 625–2704. 10.1206/377.2

[B12] Maiti P (2011) A taxonomic monograph on the world species of termites of the family Rhinotermitidae (Isoptera: Insecta). Memoirs of the Zoological Survey of India 20: 1–272.

[B13] Palin OF Eggleton P Malhi Y Girardin CAJ Rozas-Dávila A Parr CL (2011) Termite Diversity along an Amazon-Andes Elevation Gradient, Peru. Biotropica 43: 100–107. 10.1111/j.1744-7429.2010.00650.x

[B14] Pinzón OP Baquero L Beltran M (2017) Termite (Isoptera) diversity in a gallery forest relict in the Colombian eastern plains. Sociobiology 64: 92–100. 10.13102/sociobiology.v64i1.1184

[B15] Roonwal ML (1970) Measurements of termites for taxonomic purposes. Journal of Zoological Society of India 21: 9–66.

[B16] Salick J Herrera R Jordan CF Url S Salick J Jordan CF (2013) Termitaria: Nutrient patchiness in nutrient-deficient Rain Forests. Biotropica 15: 1–7. 10.2307/2387990

[B17] Silva I Vasconcellos A Moura FM (2019) Termite assemblages (Blattaria, Isoptera) in two montane forest (Brejo de Altitude) areas in northeastern Brazil. Biota Neotropica 19: 1–8. 10.1590/1676-0611-bn-2018-0519

[B18] Snyder TE (1924) An extraordinary new *Rhinotermes* from Panama. Proceedings of the Biological Society of Washington 37: 83–85.

[B19] Snyder TE (1926) Termites collected on the Mulford biological exploration to the Amazon Basin, 1991–1992. Proceedings of the US National Museum 68: 1–76. 10.5479/si.00963801.68-2615.1

[B20] Snyder TE (1949) Catalog of the termites (Isoptera) of the world. Smithsonian Miscellaneous Collections 112: 9–378.

[B21] de Souza OFF Brown VK (1994) Effects of habitat fragmentation on Amazonian termite communities. Journal of Tropical Ecology 10: 1–197. 10.1017/S0266467400007847

